# Music Therapy Self-Care Group for Parents of Preterm Infants in the Neonatal Intensive Care Unit: A Clinical Pilot Intervention

**DOI:** 10.3390/medicines5040134

**Published:** 2018-12-16

**Authors:** Esteban Roa, Mark Ettenberger

**Affiliations:** 1 Berklee College of Music, Boston, MA 02215, USA; eroafuentes@berklee.edu; 2 SONO—Centro de Musicoterapia, Bogotá 11021, Colombia; 3 Clínica de la Mujer, Bogotá 11021, Colombia

**Keywords:** music therapy, preterm infants, family-centered care, parents, self-care, wellbeing, Neonatal Intensive Care Unit (NICU)

## Abstract

**Background:** The parents of preterm infants face major mental health challenges in the Neonatal Intensive Care Unit (NICU). Family-centered music therapy actively integrates and empowers parents in their infants’ care. With the aim to better understand and address parental needs separately from their babies’ needs, a music therapy (MT) self-care group was implemented as part of clinical practice at the hospital Clínica de la Mujer, in Bogotá, Colombia. **Methods:** The group was provided for both parents, twice a week, in the NICU. Music guided relaxations, breathing techniques, and self-expression were at the center of the MT group sessions. The parents completed a pre/post self-administered Numeric Rating Scale (NRS), including anxiety levels, stress levels, mood and motivation. **Results:** The parents highly valued the MT self-care group in the NICU. On average, there was a 37% improvement in anxiety levels, 28% improvement in stress levels, and 12% improvement in mood, restfulness and motivation. Being able to relax, to distract themselves from their worries and having time for themselves are amongst the most frequently mentioned benefits. **Conclusions:** Addressing parents’ needs separately from their babies’ treatment, with culturally sensitive interventions aimed at improving parental mental health, is essential for continuing the development of family-centered music therapy interventions in the NICU.

## 1. Introduction

Having a newborn child is seen as a transformational and, often times, positive experience. However, having a preterm baby in the Neonatal Intensive Care Unit (NICU) can interrupt the transition into parenthood and cause parents to feel a surfeit of emotions, often leading to a sense of hopelessness, psychological distress, anxiety and symptoms of depression [1,2,3,4,5]. Uncertainties revolving around the infant’s health, being in an intimidating and alien environment, the financial demands of hospitalization, and the physical appearance of the infant can all be potential stressors for the parents, putting their emotional and mental wellbeing at risk [5,6,7].

Mothers of preterm infants may show long-lasting signs of posttraumatic stress, depressive symptoms, and anxiety [8,9,10]. If the trauma experienced during the perinatal period is not addressed adequately, it may negatively affect the relationship between the mother and the child, specifically the mother’s availability to recognize the infant’s cues and needs [11,12]. Recently, fathers have been taking a more active role in the NICU [13,14] and, just like mothers, fathers are susceptible to various common stressors [15]. Having to care for both their infant and their partner, the pressures of keeping up with employment obligations and possible tensions related to cultural and societal expectations regarding ‘masculinity’, or gender roles are some of the primary stressors that are specific to fathers [13,16,17,18]. Unfortunately, follow-up on parental psychological wellbeing during and after hospitalization is not always included in the neonatal care settings. There is a need for culturally appropriate interventions that take into account psychological distress and wellbeing among parents in the NICU [15,17,19,20]. Understanding and addressing parents’ needs as both connected to and distinct from their babies’ needs may help parents not only as individuals, but it may also positively impact their journey towards parenthood and the relationship they foster with their baby.

### Family-Centered Care and Family-Centered Music Therapy in the NICU

Family-Centered Care (FCC) is a growing approach to pediatric health care in which the family is recognized as the patient’s primary source of support and as a fundamental part of their wellbeing [21]. The principles of FCC include information sharing, respect and honoring differences, partnership and collaboration, negotiation, and care in the context of the family and community [22]. This approach in health care has become a fundamental pillar in contemporary neonatal care [23] and has been shown to strengthen the parent–infant relationship [9,24], increase the wellbeing of preterm infants [25], and reduce parental stress [26]. 

Music therapy in the NICU is a well-established field of clinical practice and research [27,28,29]. Current literature suggests that music therapy is beneficial for both the neonates and caregivers [30,31,32,33]. Interventions directed towards the neonate aim to promote physiological and behavioral self-regulation [31,34], feeding success [31,35,36] and improve sleep or quiet alert states [31,37]. The positive impacts of music therapy on parents include relaxation [34], the reduction of anxiety and stress levels [30,31,38], and improved parent–infant bonding [30,33,39,40,41]. Qualitative analyses show that through the use of music parents can feel empowered by having a more active role in their infants’ care [41,42,43], which is crucial for the transition into parenthood [44,45]. 

The recent shift in pediatric health care to actively integrate parents in their infants’ treatment should also consider their individual wellbeing. Family-centered music therapy in the NICU is rooted in FCC principles and stresses the importance of both infant and parental wellbeing as being essential in neonatal care settings [30,43,46,47]. In order to further develop and detect new ways to help mothers and fathers cope with potential stressors and mental health risks, a music therapy self-care group for parents was implemented as part of the ongoing clinical practice at the NICU in the Clínica de la Mujer. This article highlights the development, process, and preliminary results of this clinical pilot intervention.

## 2. Materials and Methods

### 2.1. Context and Setting

The Clínica de la Mujer is one of Bogotá’s (the capital of Colombia) most renowned maternity hospitals. Its Level-III NICU has space for 19 incubators located in individual rooms, with a few rooms reserved for twins. Parents have 24-h access to the unit and grandparents can visit the babies once a week. Kangaroo care is a standard intervention in the NICU and music therapy is part of an interdisciplinary therapy team, including respiratory therapy, speech and language therapy, nutrition, social work and psychology/psychiatry. FCC principles and a strong commitment to humanized care build the foundations of the hospital’s care philosophy. Music therapy is provided twice a week for preterm and critically ill full-term babies and their families, focused primarily on parent–infant bonding, infant self-regulation and parental mental health.

The music therapy self-care group for mothers and fathers was integrated into a music therapy clinical practice as a pilot intervention in July 2018. Initially, the idea for the group developed in response to many mothers’ concerns regarding their difficulties during breast milk extraction due to worries about their babies’ health and the pressure to produce enough milk. It was hypothesized that a music therapy self-care group could help mothers to relax and thus reduce the stress perceived during breast milk extraction. After a few pilot sessions and in discussion with the parents and the health care team, it became clear however that not only mothers but also fathers could benefit from the self-care group. As a result, the group was opened for mothers and fathers in August 2018. With the aim to improve parental wellbeing during the NICU stay, the group is provided twice a week for 15–20 min on Wednesday afternoons and for 30 min on Friday mornings.

### 2.2. Music Therapy Self-Care Group: Procedure and Interventions

The group takes place in the NICU’s breast milk extraction room before mothers are scheduled for their next time slot. Although participation in the music therapy self-care group is voluntary, the nursing and medical teams highly encourage both mothers and fathers to attend. The group usually starts with a short verbal introduction with the purpose of explaining the objectives and procedures for the new participants and to quickly assess current parents’ moods or any specific stressors.

Live music therapy is at the center of the interventions. The music therapist regularly uses a nylon-string guitar, their voice, shakers, an ocean drum, and a ‘Samafón’ as the principal instruments. The ‘Samafón’ is an instrument that is shaped similar to a lyre, but instead of the strings, five hollow metal tubes hang from the top to the base of the instrument and are connected to each other with threads. Depending on the length of the tubes, notes are lower or higher and the instrument is normally tuned in variations of the pentatonic scales. The tubes are played either individually or quickly, one after another, with a mallet used for singing bowls and the sound is long-lasting. Additionally, the instrument can be rotated easily, holding it with one hand, creating ripples of sounds that project themselves in all directions. Short musical games with shakers, voices or movements serve as ‘ice-breakers’, seeking to activate the participants at the beginning of the session. Then, the music therapist provides verbal relaxation prompts focused on deep breathing, imagery (i.e., visualizing a place where participants feel safe and comfortable, or evoking a particular landscape, such as standing on a mountain top and looking over the horizon or sitting on the beach and contemplating the movement of the waves), and/or subtle movements with the objective to foster body awareness and to make conscious current moods, thoughts, or emotions. Subsequently, live music is provided, inviting parents to breathe with the music. Alternatively, parents are encouraged to use their voices with the music by either humming or singing vowels or closed consonants in order to achieve increased lengths of exhalation and to creatively explore their musicality.

Although there is debate on what music is best for relaxation [46], the music during the interventions is typically improvised and based on elements such as a slow tempo, repetition, and subtle melodic and harmonic variations or modulations. Often, chord intervals clearly indicating major or minor tonalities (i.e., major or minor thirds) are substituted with sus^9^ or sus^4^ chords, leaving it open to the music therapist’s clinical improvisation skills to modulate between major or minor tonalities. 

Once the intervention concludes, parents verbally reflect upon their experiences, thoughts, and feelings during the group session. The music therapist discusses music-assisted self-care techniques that participants can use in their own time. Such techniques include deep breathing techniques with music or voice, the use of recorded music for relaxation, and information sharing between parents.

### 2.3. Evaluation and Measurements

**Figure 1:**
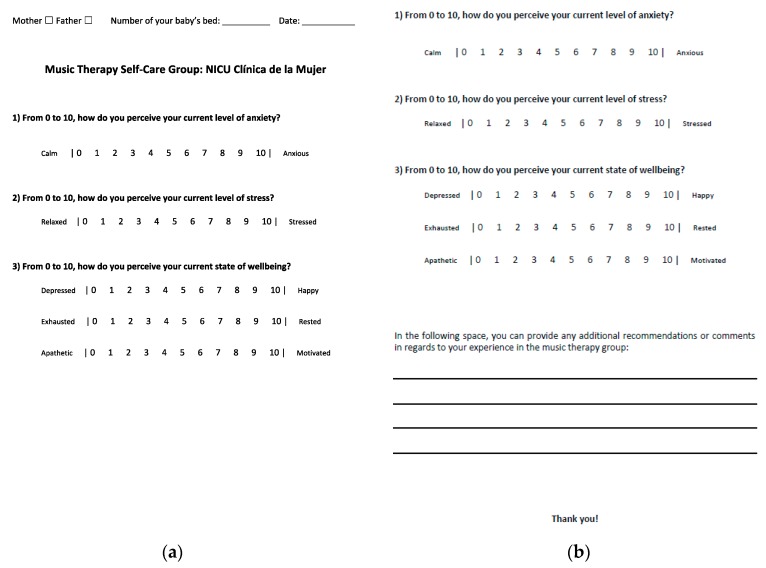
This figure shows the Numeric Rating Scale (NRS) the music therapy team designed to evaluate the clinical pilot program. (**a**) This is the pre-intervention sheet. Participants are given a few minutes prior to the intervention in order to fill it out. As shown above, the music therapy team considered anxiety, stress, and three areas of wellbeing, including mood, restfulness, and motivation. (**b**) This is the post-intervention sheet. The only difference is the ‘additional comments’ section at the bottom of the page. Participants are given a few minutes after the intervention in order to fill it out.

To evaluate the clinical pilot program and to better understand the potential benefits of the music therapy self-care group for parents in the NICU, the music therapy team designed a self-administered pre/post Numeric Rating Scale (NRS). It is important to highlight that the results gathered from the NRS are strictly used to evaluate the clinical pilot program and are not used for research purposes. The scale aims to detect changes in the perceived levels of anxiety, stress, and wellbeing before and after the music therapy intervention. An ‘additional comments’ section on the post-intervention sheet provides the opportunity for parents to share more personalized experiences and recommendations in regards to the group. Figure 1 shows the NRS that is currently used.

Parents are assured that their names and personal information are not used during the evaluation of the program. In addition to the NRS, a few semi-structured interviews were conducted with the participating parents to further understand their lived experiences with music therapy.

## 3. Results

As stated above, the music therapy self-care group took place twice a week. However, due to time restrictions on one of the days, data collection only took place before and after the thirty-minute sessions on Fridays. While the evaluation of the group is part of the clinical practice, it is hoped that the current data collection will help in the establishment of a research protocol in the near future. Table 1 shows the basic features of the group.

The results obtained with the NRS indicate a positive effect of the music therapy self-care group on parents’ perceived anxiety and stress levels, and on their mood, restfulness, and motivation. Table 2 shows the mean scores for the pre- and post-intervention.

**Figure 2:**
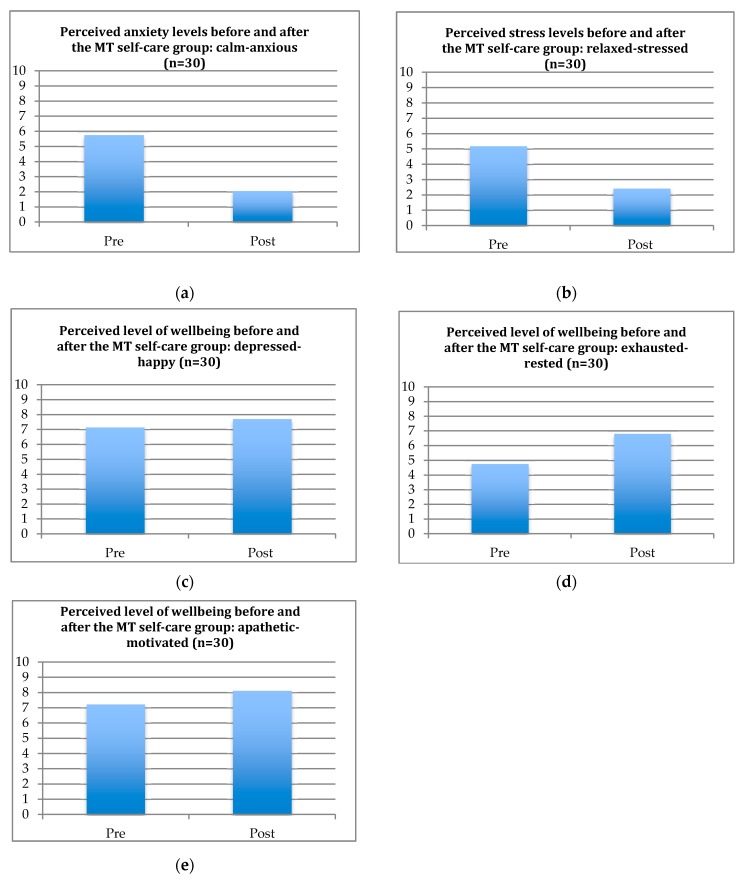
This figure shows the bar charts of the pre- and post-intervention results obtained with the NRS. Please note that the NRS was introduced at a later point, after the start of the clinical pilot program. This is why the data of 30 participants were collected via the NRS, but there were 122 participants in total. (**a**) This bar chart shows the perceived level of anxiety before and after the group sessions. A lower score indicates less perceived anxiety. (**b**) This bar chart shows the perceived level of stress before and after the group sessions. A lower score indicates less perceived stress. (**c**) This bar chart shows the perceived level of mood before and after the group sessions. A higher score indicates a better perceived mood. (**d**) This bar chart shows the perceived level of restfulness before and after the group sessions. A higher score indicates a better perceived feeling of restfulness. (**e**) This bar chart shows the perceived level of motivation before and after the group sessions. A higher score indicates a better perceived motivation.

Figure 2 shows the results as bar charts. On average, there is a 37% improvement in anxiety levels, a 28% improvement in stress levels, a 6% improvement in mood, a 20% improvement in restfulness, and a 9% improvement in motivation. Being able to relax, to distract themselves from their worries and to have time for themselves are amongst the most frequently mentioned benefits parents left in the ‘additional comments’ section.

## 4. Limitations

Although the evaluation of the music therapy self-care group was not part of the research protocol, the clinical pilot intervention faced the following limitations that should be considered for future investigations:

- The sessions take place in the breast milk extraction room between scheduled milk extraction time slots. Due to its size, it is currently the only available space in the NICU appropriate for group interventions. However, since breast milk extraction usually takes place continuously throughout the day, this caused difficulties with respect to the time restriction for the group on one of the days (Wednesday afternoon).
- The clinical pilot program aims to address the needs of both mothers and fathers. Bearing in mind the cultural norms, perceptions on gender roles, and employment obligations in Colombian society, there were not as many fathers present as mothers throughout the pilot program. Nonetheless, participating fathers regularly express having a positive experience with the group and encouraged that it be formalized in NICU care.
- Since mothers do not always schedule the same time slot for breast milk extraction and kangaroo care with their babies, participation in the group session was inconsistent. Some mothers joined the group several times, but others participated just once. To better assess the outcomes, it is suggested that the participants have a consistent and scheduled attendance for the music therapy self-care group.
- The music therapy team designed the NRS which was used to evaluate the clinical pilot intervention based on the experiences in clinical practice; it is not a standardized assessment tool and has not been validated. Nevertheless, it seems to be an intuitive and easily applicable tool to quickly assess some of the most important domains of parental wellbeing and the parents reported no difficulties in understanding the NRS.

## 5. Discussion and Conclusions

Parents in the NICU face many challenges that may have potential negative and long-lasting mental health implications. These challenges put not only the individual wellbeing of mothers and fathers at risk, but might also affect the evolving relationship with their baby [1,10,11,12,48,49]. Parents frequently communicate the need to access tools to better cope with the difficulties that a hospitalization in the NICU can imply and those parents who are provided with appropriate strategies feel empowered, which in turn can positively affect their personal wellbeing and their babies’ development [50].

Despite the recent shift in NICU music therapy towards a more inclusive approach of mothers and fathers, studies that focus on relevant parental outcomes are still scarce [51]. Parental wellbeing is also influenced by many different factors, making it difficult to define and measure [52]. The music therapy self-care group at the Clínica de la Mujer was implemented to consciously try to address some of the most common parental stressors mentioned in pediatric health care literature [50] and to focus on parents’ needs as individuals. The sessions aimed at providing a space for parents to work on their personal wellbeing using simple and transferable tools relevant for resilience and coping. The results gathered from the NRS are promising and indicate that mothers and fathers experience a decrease in perceived levels of anxiety and stress, and an improvement in mood, relaxation and restfulness after the intervention.

*“A state of wellbeing is easily achieved with music therapy. Being in the NICU is stressful, but it becomes tolerable with this type of support.”*

*(A participating mother)*

Although no data were collected regarding our initial motive to reduce maternal stress and anxiety during breast milk extraction, a limited number of articles report a positive impact of recorded music on breast milk quantity and quality [53,54]. An improved relaxation response in mothers has been discussed as a potential mechanism in both of the aforementioned studies, and is in-line with the already documented anxiolytic and stress-reducing effects of music and music therapy interventions in the NICU [27,28,29,30,34,55,56]. Thus, a positive impact of the music therapy self-care group on maternal breast milk extraction seems to be a feasible hypothesis that needs to be investigated with a formal research protocol in the future.

*“It is a great space because it allows us to rest and disconnect from the situation we are going through.”*

*(A participating father)*

For this clinical pilot intervention, live music was considered the best practice due to the flexibility and ability to adapt the music depending on the parents’ responses and current needs. Moreover, live music allows for a group dynamic in which participants can interact and share their experiences with one another. Considering that information sharing is at the core of the FCC principles [21,22], the music therapist regularly encourages and facilitates participants to share their experiences after the intervention and to use the space to build a support system amongst themselves. The time allocated for reflection and discussion after the intervention allows parents to share not only their current struggles and difficulties, but also ways to cope with them. Furthermore, considering the ‘collective’ nature of Colombian society, the active and interactive participation of parents was considered to be more culturally appropriate than individually using recorded music.

*“It is a relaxing activity. It should be done more frequently. It allows us to alleviate the stress from this situation.”*

*(A participating mother)*

This article highlights the impact of a music therapy self-care group on parental wellbeing in the NICU. The preliminary findings from this clinical pilot intervention indicate that this may be an appropriate intervention to help parents with stress reduction, coping, and relaxation. The pilot program aims to develop a research protocol in the near future to better evaluate the impact of the music therapy self-care group on parental mental health and on mothers’ breast milk extraction. Addressing parents’ needs separately from their babies’ treatment, with culturally sensitive interventions aimed at improving parental mental health, is essential for continuing the development of family-centered music therapy in the NICU.

## Figures and Tables

**Table 1:** Basic features of the music therapy self-care group since its implementation in July 2018 to November 2018.

Total Number of Sessions	Total Number of Participants	Total Number of Mothers	Total Number of Fathers
30	122	106	16

**Table 2:** The mean scores for anxiety and stress levels, mood, restfulness and motivation before and after the music therapy self-care group.

Intervention	Anxiety Levels	Stress Levels	Mood	Restfulness	Motivation
Pre-intervention	5.8	5.2	7.1	4.8	7.2
Post-intervention	2.1	2.4	7.7	6.8	8.1
